# Entropy Production in Epithelial Monolayers Due to Collective Cell Migration

**DOI:** 10.3390/e27050483

**Published:** 2025-04-29

**Authors:** Ivana Pajic-Lijakovic, Milan Milivojevic

**Affiliations:** Faculty of Technology and Metallurgy, Belgrade University, Karnegijeva 4, 11000 Belgrade, Serbia; mmilan@tmf.bg.ac.rs

**Keywords:** viscoelasticity caused by collective cell migration, accumulation of mechanical stress, cell-cell interactions, contact inhibition of locomotion, cell jamming state transition

## Abstract

The intricate multi-scale phenomenon of entropy generation, resulting from the inhomogeneous and anisotropic rearrangement of cells during their collective migration, is examined across three distinct regimes: (i) convective, (ii) conductive (diffusion), and (iii) sub-diffusion. The collective movement of epithelial monolayers on substrate matrices induces the accumulation of mechanical stress within the cells, which subsequently influences cell packing density, velocity, and alignment. Variations in these physical parameters affect cell-cell interactions, which play a crucial role in the storage and dissipation of energy within multicellular systems. The internal dynamics of entropy generation, as a consequence of energy dissipation, are characterized in each regime using viscoelastic constitutive models and the surface properties at the cell-matrix biointerface. The focus of this theoretical review is to clarify how cells can modulate their rate of energy dissipation by altering cell-cell and cell-matrix adhesion interactions, undergoing changes in shape, and re-establishing polarity due to the contact inhibition of locomotion. We approach these questions by discussing the physical aspects of these complex phenomena.

## 1. Introduction

Entropy is one of the most fascinating concepts in physics and it plays a crucial role in quantifying the irreversibility of change in thermodynamic systems, which is a consequence of the energy dissipation that takes place during various processes [1,2,3]. The universality of this concept, its applicability to irreversible physical processes both near to and far from equilibrium as well as its connection to statistics, highlights entropy as a bridge between natural sciences and philosophy [4]. Self-maintenance within a set of catalytic chemical reactions and conversion of biochemical energy into mechanical energy, and the reverse process, occur through intricate molecular interactions between an organism and its environment [5,6]. Living cells are profoundly affected by their microenvironment, which alters their mechanical properties and structural composition, and most importantly, their metabolic activities [7]. This energy self-generation, as well as conversion, and transport, are fundamental to the emergence and maintenance of life, including morphogenesis and wound healing occurring over a range of time scales. Energy conversion and transport can be optimised by reducing energy dissipation. Peter McClintock [8] pointed out that a structural organisation capable of supporting the network of molecular processes responsible for controllable production and utilization of energy is a necessary condition for life. In accordance with the fact that life represents a “battle for structural ordering” [9], the emergence of life and its evolution can be discussed in the context of entropy production.

Alterations in energy transformation, coupled with induced energy dissipation, can result in diverse mutations and the emergence of cancerous diseases [10]. This aligns with the understanding that energy dissipation is primarily an undesirable loss of energy; however, within biochemical processes, it has the potential to modify biochemical pathways and promote the advancement of cancer [11,12]. As a result, numerous therapeutic approaches have been developed to enhance metabolic energy dissipation in tumours [12]. Cells possess the ability to regulate energy dissipation to a certain degree through mechanisms of cell signalling and gene expression, which in turn influence the remodelling of adhesion contacts between cells and the extracellular matrix, as well as alterations in cell morphology and division [13,14,15,16].

The main goal of this review is to emphasize the impact of the viscoelasticity of migrating epithelial collectives on the dissipation of mechanical energy and entropy production. Collective cell migration is an integral part of many biological processes such as morphogenesis, wound healing, and the spreading of cancer. Consequently, a persistent migration of aligned cells, quantified by the velocity correlation length and the persistence of cell migration, can reduce energy dissipation and protect living organisms against various mutations and the development of cancer [17,18]. This complex phenomenon will be discussed for model systems such as the collective migration of epithelial monolayers on substrate matrices. Migrating epithelial collectives can be treated as irreversible thermodynamic systems far from equilibrium. The multi-scale nature of entropy production is influenced by the viscoelasticity and surface characteristics of multicellular systems, particularly in relation to the epithelial and matrix surface tensions, as well as the interfacial tension that exists between them [19]. Entropy production at the supracellular level, which takes place over a time scale of hours, is examined through the lens of continuum mechanics, particularly in relation to thermodynamic entropy as influenced by cellular velocity, packing density, and alignment. In contrast, entropy production at the cellular level is analysed in terms of conformational entropy, which occurs over a time scale of minutes. The viscoelastic behaviour, in conjunction with the surface properties of epithelial monolayers, is contingent upon the interactions between cells, and between cells and the matrix, which have a feedback effect on cell migration [19,20]. In this context, three regimes of cell migration will be discussed depending on the interplay between the cell packing density, cell velocity, and degree of cell orientation in the direction of migration. They are the convective, conductive (diffusion), and sub-diffusion regimes. Our analysis will focus on energy dissipation and entropy production, drawing upon a range of experimental data from the existing literature, complemented by considerations from multi-scale modelling.

## 2. Phenomenological Description of Migrating Epithelial Monolayers on Substrate Matrices

**Figure 1:**
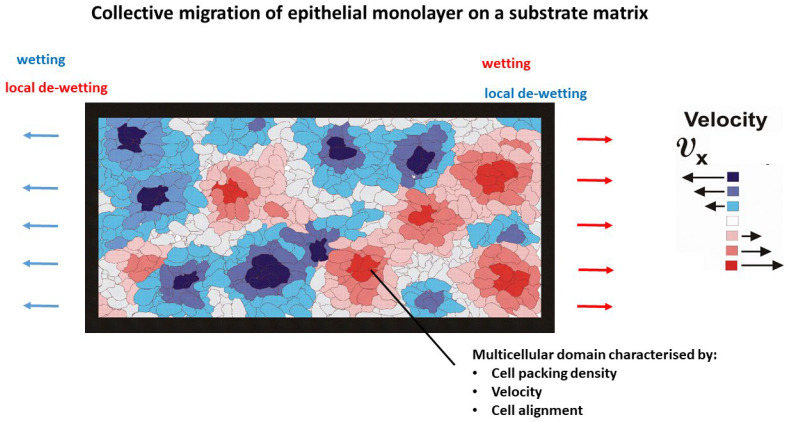
Collective migration of an epithelial monolayer on a substrate matrix, presented as an ensemble of multicellular domains. This figure is inspired by experimental data from Serra-Picamal et al. [21]. Various colours represent various velocities and directions of migration of multicellular domains. Blue domains move to the left-hand side, while red domains move to the right-hand side.

We focus here on internal entropy production during cell rearrangement caused by the collective migration of epithelial monolayers on substrate matrices. The main characteristics of migrating epithelial collectives are the inhomogeneous distribution of cell packing density, degree of cell orientation in the direction of cell migration, cell velocity, corresponding strain, and mechanical stress [19,20,21,22,23]. Cell packing density and cell velocity vary within collectively migrating epithelial monolayers. Tlili et al. [23] investigated the active wetting properties of MDCK epithelial monolayers and found that the cell packing density ranged from ${1\times 10}^{5}\;\frac{cells}{{cm}^{2}}$ to ${5\times 10}^{5}\;\frac{cells}{{cm}^{2}}$. An increase in cell packing density from ${1\times 10}^{5}\;\frac{cells}{{cm}^{2}}$ to ${5\times 10}^{5}\;\frac{cells}{{cm}^{2}}$ resulted in a decrease in cell velocity from $0.8\;\frac{\mu m}{min}$ to zero [23]. Similarly, Nnetu et al. [22] noted that the velocity of breast epithelial MCF-10A cells reached zero at a packing density of approximately $\sim {3.5\times 10}^{5}\;\frac{cells}{{cm}^{2}}$. indicating a phenomenon known as cell jamming (Box 1 and Box 2). This suggests that multicellular systems can be conceptualised as an ensemble of multicellular domains as shown in Figure 1.

The domains satisfy the following conditions:

- Every domain represents a canonical ensemble of cells and can be described by homogeneous distributions of cell packing density, cell velocity, degree of cell orientation in the direction of movement, and cell mechanical stress.
- The lifetime of domains is on a time scale of hours.
- Vanishing of the domains is caused by (i) domain active and passive wetting/de-wetting and (ii) collisions between neighbouring domains.

The domain viscoelasticity and surface characteristics, which depend on cell packing density, and cell velocity have an impact on the internal production of entropy. This is consistent with the fact that a change in cell packing density influences cell-cell interactions by changing intra-cellular distances and cellular alignment in the direction of migration [20,24].

Box 1. **The main characteristics of cell-cell interactions.**

Two types of cell-cell interaction have been identified in the context of collective cell migration: positional and orientational interactions, which are interrelated [16,25]. Positional interactions among cells are influenced by the stretching or compression of adhesion contacts mediated by E-cadherin. As a result, the interaction energy can exhibit repulsive characteristics when the local distance between cells is less than approximately 8 μm, while it becomes attractive at greater distances [26]. The characteristic time for conformational changes of proteins such as E-cadherin and integrin that represent integral parts of cell-cell and cell-matrix adhesion contacts as well as the turnover time of these proteins corresponds to a time scale of minutes [27].

Cell-cell orientational interactions depend on the angle between neighbour cells. These interactions are influenced by compressive and shear stress components capable of inducing topological defects of cell alignment, pronounced in overcrowded environments [28]. Two primary interaction types, namely head-on and glancing interactions, play a crucial role in the rearrangement of cells [20,29,30]. Head-on interactions initiate cell re-polarisation, which is associated with a reduction in both cell-cell and cell-matrix adhesion contacts, a phenomenon referred to as contact inhibition of locomotion [29]. After re-polarisation, cells establish cell-cell and cell-matrix adhesion contacts and start migration in the opposite direction to each other. In contrast, glancing interactions do not facilitate cell repolarization; however, they induce cell rotation during the process of realignment, potentially leading to the disruption of cell-matrix adhesion contacts [19]. The cell re-polarisation occurs on a time scale of hours [31].

The cell packing density within migrating epithelial monolayers varies from $n_{e}\leq n_{conf}$ to $n_{e}\to n_{j}$ (where $n_{e}$ is the cell packing density, $n_{conf}$ is the cell packing density in the confluent state, and $n_{j}$ is the cell packing density in the cell jamming state). Petitjean et al. [32] demonstrated that the Madin Darby Canine Kidney (MDCK) cell monolayers attained confluence at a cell packing density of approximately $n_{conf}\sim 2.5\times 10^{5}\;\frac{cells}{{cm}^{2}}$ and a cell velocity of $\sim 0.14\;\frac{\mu m}{min}$. The cell packing density $n_{j}$ is a few times higher than the cell packing density in the confluent state [22]. The corresponding cell speed $\left\|{\vec{v}}_{e}\right\|$ decreases with the cell packing density from $0.1<\left\|{\vec{v}}_{e}\right\|<\sim 1\;\frac{\mu m}{\min}$ to $\left\|{\vec{v}}_{e}\right\|\to 0$ for cells in the jamming state.

Box 2. **The cell jamming state transition: the main physical characteristics.**

The transition to a jamming state in a collection of cells denotes a change from a contractile to a non-contractile state, induced by contact inhibition of locomotion. This effect is particularly significant in overcrowded environments where there is high compressive stress [33,34]. The contact inhibition of locomotion, caused by cell orientational, head-on interactions (Box 1), triggers the mechanism of cell re-polarisation by weakening the cell-cell and cell-matrix adhesion contacts [29]. In the context of cell response to head-on interactions, two characteristic times should be introduced, i.e., the necessary time for cell re-polarisation, and the time between two successive cell collisions. Notbohm et al. [31] revealed that the average re-polarisation time of MDCK cell monolayers is 1.28 h. When cells do not have enough time to re-polarise between two collisions, they enter the non-contractile (jamming) state, while the cell velocity drops to zero [34]. The jamming state transition can be treated as a rigidity state transition [34]. This aligns with the observation that active (contractile) cells exhibit significantly greater stiffness compared to passive (non-contractile) cells, a phenomenon attributed to the accumulation of contractile energy. Research by Schulze et al. [35] indicates that Young’s modulus for contractile MDCK cell monolayers is approximately 33.0 ± 3.0 kPa, whereas the modulus for non-contractile cells is approximately half of this value. A reduction in cell-cell adhesion contacts results in energy dissipation, which subsequently decreases the compressive stress. As a result, cells experience an unjamming transition and resume migration.

**Figure 2:**
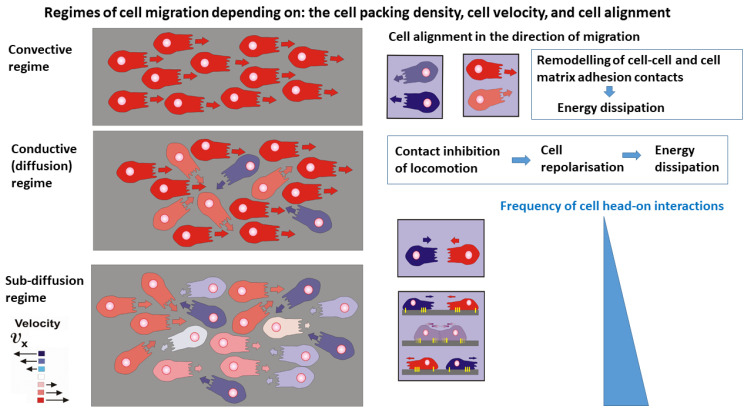
Schematic presentation of various regimes of cell migration depending on the cell packing density, cell velocity, and order parameter. Red and blue cells are members of forward and backward flows caused by partial de-wetting. Pink and grey cells have lower velocities than red and blue ones, while white cells are in the resting (non-contractile) state. An increase in cell packing density caused by the accumulation of cell compressive stress intensifies contact inhibition of locomotion responsible for the long-term energy dissipation within diffusion and sub-diffusion regimes. Remodelling of cell-cell and cell-matrix adhesion contacts during cell alignment induces the short-term energy dissipation characteristic for a convective regime.

Consequently, the production of entropy can be discussed within three regimes of cell migration: the convective, diffusion, and sub-diffusion regimes as shown in Figure 2:

The main characteristics of the regimes are given as follows:

- The convective mechanism of cell migration arises within domains that satisfy the conditions that the cell packing density is $n_{e}\leq n_{conf}$ and the cell speed is $0.1<\left\|{\vec{v}}_{e}\right\|<\sim 1\;\frac{\mu m}{\min}$. The velocity correlation length, i.e., the distance over which cells move in a correlated fashion, is about 10 cell lengths [17], indicating an anisotropic behaviour. Contact inhibition of locomotion, caused by cell orientational interactions, is rare. Energy dissipation is caused primarily by the remodelling of cell-cell and cell-matrix adhesion contacts that occurs on a time scale of minutes [19].
- The conductive (diffusion) mechanism of cell migration arises within domains of higher cell packing density, i.e., for cell packing density $n_{j}>n_{e}>n_{conf}$ and $\left\|{\vec{v}}_{e}\right\|\sim 10^{-3}-10^{-2}\frac{\mu m}{\min}$ (where $n_{j}$ is the cell packing density under cell jamming). An increase in cell packing density perturbs the cell alignment, quantified by a decrease in the velocity correlation length [17,32]. This regime of cell packing densities could be considered as an isotropic cell rearrangement. The perturbation of cell alignment leads to an increase in contact inhibition of locomotion [25,29,30]. Altered cell re-polarisation accompanied by a weakening of cell-cell and cell-matrix adhesion contacts is the main cause of energy dissipation in this regime [29,30]. Consequently, energy dissipation occurs on a time scale of hours. The main characteristic of this regime is that the re-polarisation time is shorter than the time between collisions, which enables cells to finalise the re-polarisation process and continue migration in the opposite direction by preventing cell jamming.
- The damped conductive (sub-diffusion) mechanism occurs under high cell packing density near cell jamming within domains satisfying the conditions that $n_{e}\to n_{j}$ and $\left\|{\vec{v}}_{e}\right\|\to 0$. The velocity correlation length corresponds to the length of a single cell [17] indicating isotropic cell rearrangement. This high cell packing density causes intensive cell-cell interactions leading to collective contact inhibition of locomotion (Box 1). However, in contrast to the previous case, the time necessary for cell re-polarisation is longer than the time between two cell collisions and cells are unable to finalise the re-polarisation process. The cells then undergo jamming. Consequently, energy dissipation within cell collectives near jamming occurs on a time scale of hours.

The production of entropy, induced by energy dissipation, occurs on two time scales. Cell shape changes and the remodelling of cell-cell and cell-matrix adhesion contacts occur on a time scale of minutes. Cell alignment, re-polarisation, and velocity change accompanied by the corresponding strain, and cell residual stress accumulation occur on a time scale of hours. Cell division in various types of epithelial cells typically occurs over a time frame of several days and is further extended in overcrowded environments leading to some parts of a multicellular system being in the quiescent (non-proliferative) state [36,37,38].

In the discussion below, we will explore the connection between long-term entropy production and the energy dissipation rate associated with collective cell migration, incorporating relevant physical parameters. The dissipation of energy is influenced by the viscoelastic properties and surface characteristics of epithelial monolayers, which will be examined within the context of the defined regimes of cell migration.

## 3. Long Term Change of Internal Entropy Caused by Collective Cell Migration

A migrating epithelial collective can be treated as a closed thermodynamic system far from equilibrium. The energy storage, caused by cell rearrangement, can be expressed in the form of the Helmholtz free energy $F_{e}\left(r,\tau \right)=U_{e}-T\;S$ (where $U_{e}$ is the internal energy of the multicellular system, *T* is the temperature, which is about 300 K for biological systems and S is the entropy). The internal energy of multicellular systems includes cumulative effects of various biochemical processes influenced by cell contractions and crosstalk between cell-cell and cell-matrix adhesion contacts, which occur through cytoskeleton rearrangement. An isothermal change of the free energy can be expressed as $\frac{dF_{e}\left(r,\tau \right)}{d\tau }=\frac{dU_{e}\left(r,\tau \right)}{d\tau }-T\frac{dS\left(r,\tau \right)}{d\tau }$. The internal energy change can be expressed from the first law of thermodynamics as $\frac{dU_{e}\left(r,\tau \right)}{d\tau }=\frac{\delta Q}{\delta \tau }-\frac{dW_{e}}{d\tau }$ (where Q is a heat primarily generated by frictional effects and $W_{e}$ is the work of cell rearrangement $W_{e}={\tilde{\sigma }}_{re}:{\tilde{\varepsilon }}_{e}$**,** ${\tilde{\sigma }}_{re}$ is the cell residual mechanical stress generated during collective cell migration, and ${\tilde{\varepsilon }}_{e}$ is the corresponding cell strain). The entropy change can be expressed based on the second law of thermodynamics as $\frac{dS}{d\tau }=\frac{1}{T}\frac{\delta Q}{\delta \tau }+\frac{dS_{rear}}{d\tau }$ (where $\frac{dS_{rear}}{d\tau }$ is the internally generated entropy during collective cell migration, which satisfies the condition for irreversible processes that $\frac{dS_{rear}}{d\tau }>0$). Consequently, the production of entropy can be expressed as $\frac{dS_{rear}}{d\tau }=\frac{1}{T}\left(\frac{dW_{e}}{d\tau }-\frac{dF_{e}\left(r,\tau \right)}{d\tau }\right)$ (where $\frac{dW_{e}}{d\tau }-\frac{dF_{e}\left(r,\tau \right)}{d\tau }$ represents the rate of energy dissipation $\frac{dW_{d}}{d\tau }=\frac{dW_{e}}{d\tau }-\frac{dF_{e}\left(r,\tau \right)}{d\tau }$).

Long-term entropy production depends on the viscoelasticity and surface characteristics of multicellular systems, while the viscoelasticity itself relates to cell velocity, packing density, and cell alignment in the direction of migration. It can be expressed under isothermal conditions as

(1) $$\frac{dS_{i}\left(r,\tau \right)}{d\tau }=\frac{1}{T}\frac{dW_{d}\left(r,\tau \right)}{d\tau }$$

where T is the temperature and $W_{d}=W_{d}\left({\vec{v}}_{e},\langle \vec{Q}\rangle ,n_{e}\right)$ is the energy dissipation, while ${\vec{v}}_{e}$ is the cell velocity, $\langle \vec{Q}\rangle$ is the average orientational vector within the domain expressed as $\langle \vec{Q}\rangle \left(r,\tau \right)=\sum_{i}{\vec{Q}}_{i}\delta \left(r-r_{i}\left(\tau \right)\right)$, and $n_{e}$ is the cell packing density). Consequently, the local internal entropy production during cell rearrangement can be expressed as

(2) $$\frac{dS_{rear}\left(r,\tau \right)}{d\tau }={\left(\frac{\partial S_{rear}}{\partial n_{e}}\right)}_{\left\|{\vec{v}}_{e}\right\|,\left\|\langle \vec{Q}\rangle \right\|}\frac{dn_{e}}{d\tau }-{\left(\frac{\partial S_{rear}}{\partial \left\|{\vec{v}}_{e}\right\|}\right)}_{n_{e},\left\|\langle \vec{Q}\rangle \right\|}\frac{d\left\|{\vec{v}}_{e}\right\|}{d\tau }-{\left(\frac{\partial S_{rear}}{\partial \left\|\langle \vec{Q}\rangle \right\|}\right)}_{\left\|{\vec{v}}_{e}\right\|,n_{e}}\frac{d\left\|\langle \vec{Q}\rangle \right\|}{d\tau }$$

where $S_{rear}$ is the local entropy caused by cell rearrangement and $\frac{dS_{rear}\left(r,\tau \right)}{d\tau }$ is the rate of entropy production. The entropy production rate increases with increasing cell packing density $n_{e}$ and decreases with an increase in cell speed $\left\|{\vec{v}}_{e}\right\|$ and the degree of cell orientation in the direction of migration (the order parameter) $\left\|\langle \vec{Q}\rangle \right\|$. The order parameter $\left\|\langle \vec{Q}\rangle \right\|$ ranges from 0 for a completely disordered multicellular domain, to 1 for a domain that is completely aligned [39]. Consequently, the parameter $\left\|\langle \vec{Q}\rangle \right\|$ can be treated as a measure of the domain anisotropy such that $\left\|\langle \vec{Q}\rangle \right\|=0$ corresponds to isotropic viscoelasticity, while the degree of anisotropy increases with $\left\|\langle \vec{Q}\rangle \right\|$. For $\left\|\langle \vec{Q}\rangle \right\|\to 1$ the viscoelasticity is totally anisotropic.

The evidence suggests that (1) the cell speed oscillates [31,40], (2) variations in cell packing density result from oscillations in cell normal stress (Box 3) [19,28], (3) cell normal stress exhibits long-term oscillatory behaviour [21,31], and (4) the order parameter undergoes fluctuations due to repeated perturbations and realignments of cell orientation [28,41]. Consequently, it can be inferred that the internal production of entropy $\frac{dS_{rear}\left(r,\tau \right)}{d\tau }$ also undergoes oscillations.

Box 3. **Oscillations of physical parameters: mechanical waves.**

Collective cell migration generates mechanical waves, as noted by Serra-Picamal et al. [21] and Notbohm et al. [31], which have been examined in relation to low Reynolds turbulence [42]. These mechanical waves are characterized by sustained oscillations in cell velocity, associated strain, and mechanical stress, and they have been analysed in terms of effective inertia [26,31,42]. The oscillatory nature of mechanical stress results in variations in cell packing density: (i) compressive stress leads to an increase in cell packing density, (ii) tensional stress results in a decrease in cell packing density, and (iii) shear stress does not affect cell packing density.

**Figure 3:**
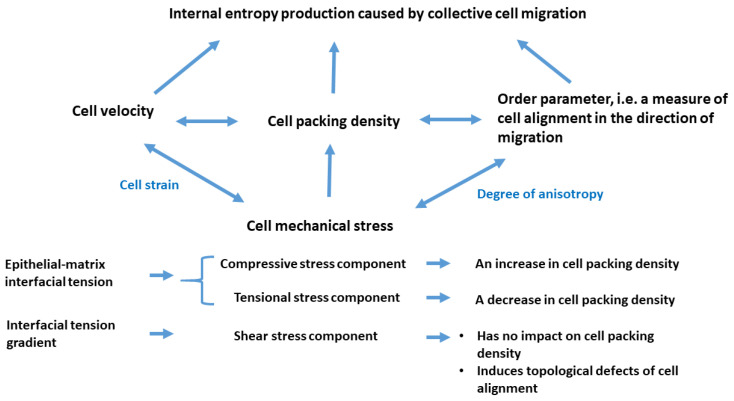
Relationship between the physical parameters that contribute to entropy production.

The relationship between long-term entropy production, energy dissipation, and relevant physical parameters is shown schematically in Figure 3.

The set of physical parameters established depends on the cumulative effects of cell-cell and cell-matrix interactions, which have a feedback impact on the surface characteristics of epithelial collectives, quantified by epithelial cohesiveness and epithelial-matrix adhesiveness, and viscoelasticity caused by collective cell migration [19,20]. These parameters will be formulated and discussed in the next two sections.

### 3.1. Surface Characteristics of Epithelial Monolayers

The cohesion and adhesion energies of multicellular systems depend on the strength of cell-cell and cell-matrix adhesion contacts and on the crosstalk between the adhesion contacts, managed by the cytoskeleton contractions [13]. The strength of cell-cell and cell-matrix adhesion contacts relies on cell-cell positional and orientational interactions depending on micro-environmental conditions such as the stiffness and rheological behaviour of the extracellular matrix and cell packing density [16,20]. An inhomogeneous distribution of cell packing density accompanied by mechanical stress induces an inhomogeneous distribution in the strength of cell-cell adhesion contacts [26,43].

An interrelation between cohesion and adhesion energies, expressed in the form of the spreading factor is responsible for cell active or passive wetting (extension)/de-wetting (compression) of epithelial monolayers on substrate matrices [19,43]. Active wetting/de-wetting occurs via collective cell migration, while passive wetting/de-wetting is driven by the gradient of the epithelial matrix interfacial tension [19]. This phenomenon, known as the Marangoni effect [44], significantly influences cell rearrangement and the generation of cell shear stress. The spreading factor accompanied by other physical parameters included in the surface characterization of epithelial monolayers is shown in Table 1.

The contractile properties of epithelial cells contribute to the reinforcement of cell-cell adhesion interactions, thereby enhancing the epithelial surface tension [50]. Furthermore, the expansion of epithelial monolayers through collective cell migration results in an increase in epithelial surface tension [51]. Conversely, mechanical compression may disrupt cellular alignment, potentially weakening E-cadherin-mediated adhesion and leading to a reduction in epithelial surface tension [48]. The variation in the extension of certain multicellular domains, in contrast to the compression observed in others, leads to an inhomogeneous distribution of epithelial surface tension and its long-term change. The matrix surface tension $\gamma_{m}$ depends on the flexibility of the polymer chains and the strength of the inter-chain bonds. Cell tractions can induce the disordering of chain organization and the establishment of local polymer concentration gradients, which have a feedback impact on the distribution of the matrix surface tension [48,52]. The gradient of the matrix surface tension is one of the driving forces for directional cell migration [48]. The cell-matrix adhesion energy $e_{a}$ depends on the strength of cell-matrix adhesion contacts and on that basis relies on matrix stiffness, cell velocity, and packing density. Non-contractile cells establish weak cell-matrix adhesion contacts. An increase in adhesion energy $e_{a}$ leads to a reduction of epithelial-matrix interfacial tension (Table 1). The inhomogeneous distribution of epithelial and matrix surface tension accompanied by cell-matrix adhesion energy causes an inhomogeneous distribution of the interfacial tension and generation of the interfacial tension gradient [19,49]. The interfacial tension gradient drives the passive extension of multicellular domains from the regions of lower interfacial tension to the regions of higher interfacial tension [19].

As a result, both the interfacial tension and its gradient play a significant role in the generation of the cell residual stress. In multicellular systems, residual stress is maintained during collective cell migration and shows variations over a period of several hours [19]. The cell’s normal residual stress consists of isotropic and deviatoric parts. The isotropic part of the stress is caused by the work of cell-matrix interfacial tension expressed by the Young–Laplace equation. The interfacial tension does work along the biointerface area between the epithelial monolayer and substrate matrix in order to reduce the interface area [48]. The interfacial tension is, therefore, a key factor influencing the compression and extension of an epithelial monolayer as well as the surrounding matrix. When the epithelial monolayer undergoes extension during its movement, commonly referred to as cell wetting, this process induces compression of the underlying matrix [48]. Conversely, when the monolayer experiences compression due to de-wetting, it results in the relaxation of matrix strain in the region of the cell-matrix biointerface, which can be characterized as an expansion. Compression of an epithelial monolayer is labelled with the sign “+”, while its extension is labelled with a “−“. The deviatoric part of cell normal residual stress is induced by collective cell migration. Consequently, the cell’s normal residual stress can be expressed as

(3) $$\sigma_{ri=1/2}=\pm ∆p_{c\to m}\pm {\sigma_{ri}}^{CCM}$$

where the subscript i≡xx,yy for the normal stress (xx≡1, yy≡2), $∆p_{c\to m}$ is the isotropic part of the cell’s normal stress equal to $∆p_{c\to m}=-\gamma_{em}\left(\vec{𝜵}\cdot \vec{n}\right)$, $\vec{n}$ is the normal vector of the cell-matrix biointerface, and ${\sigma_{ri}}^{CCM}$ is the normal cell residual stress component caused by collective cell migration. The shear residual stress component accounts for two contributions. One contribution is induced by the interfacial tension gradient and described as natural convection, while the other contribution is caused by collective cell migration and described as forced convection [48]. Consequently, the shear residual stress component is expressed as

(4) $$\sigma_{ri=3}=\vec{𝜵}\gamma_{em}\cdot \vec{t}+{\sigma_{ri}}^{CCM}$$

where the subscript 3≡xy=yx and $\vec{t}$ is the tangent vector of the epithelial-matrix biointerface. The first term of the right-hand side represents the shear stress contribution caused by natural convection, while the second term represents the contribution caused by forced convection, i.e., collective cell migration.

Energy dissipation caused by cell rearrangement via collective cell migration is expressed here as

(5) $$W_{d}\left(r,\tau \right)={W_{d}}^{surf}\left(\gamma_{em},\vec{𝜵}\gamma_{em}\right)+W_{d}^{CCM}$$

where ${W_{d}}^{surf}$ is the energy dissipation caused by the change of the interfacial tension, equal to ${W_{d}}^{surf}\left(r,\tau \right)=\sum_{i=1}^{3}\sigma_{i}^{surf}\varepsilon_{i}$, such that the normal stress components satisfy the condition of isotropy, i.e., $\sigma_{1}^{surf}\left(\gamma_{em}\right)=\sigma_{2}^{surf}\left(\gamma_{em}\right)=∆p_{c\to m}$ (Equation (3)), while $\sigma_{3}^{surf}\left(\vec{𝜵}\gamma_{em}\right)$ is the shear stress component (Equation (4)), $\varepsilon_{i}$ are strain components such that $\varepsilon_{1}$ and $\varepsilon_{2}$ are normal strain components equal to $\varepsilon_{1/2}\left(r,\tau \right)=\frac{\partial u_{k}}{\partial k}$, (k≡xx,yy), and $\varepsilon_{3}$ is the shear strain component equal to $\varepsilon_{3}\left(r,\tau \right)=\frac{1}{2}\left(\frac{\partial u_{k}}{\partial l}+\frac{\partial u_{l}}{\partial k}\right)$**,** (k≡x and l≡y), $u_{i}$ are components of the epithelial displacement vector $\vec{u}\left(r,\tau \right)$, while the epithelial velocity represents the rate of change the epithelial displacement vector, i.e., ${\vec{v}}_{e}\left(r,\tau \right)=\frac{d\vec{u}}{d\tau }$, and $W_{d}^{CCM}$ is the energy dissipation caused by collective cell migration.

Energy dissipation $W_{d}^{CCM}$, as a product of the viscoelasticity of a multicellular system, depends on the regime of cell migration. Three regimes have been observed experimentally: convective, conductive (diffusion), and sub-diffusion depending on cell speed and cell packing density [21,22,31,53]. It is necessary to propose a suitable constitutive model of viscoelasticity for each regime and then to formulate this contribution to the energy dissipation.

### 3.2. Viscoelasticity of Epithelial Monolayers: The Energy Dissipation

Each regime of cell migration is related to a particular constitutive model of viscoelasticity described as shown in Table 2.

where the subscripts i,j≡xx,yy, xy; (xx≡1, yy≡2, xy≡3), $c_{ij}$ are stiffness constants, $\eta_{ij}$ are anisotropic viscosities, $\tau_{Rc}$ is the cell stress relaxation time, $E_{i}$ is the elastic modulus, $\eta_{i}$ is the cell isotropic viscosity (shear or bulk), r is the space coordinate, t is a short-time scale (i.e., minutes), τ is a long-time-scale (i.e., hours), $\left\|{\vec{v}}_{e}\right\|$ is the cell speed, ${\vec{v}}_{e}$ is the cell velocity equal to ${\vec{v}}_{e}=\frac{d\vec{u}}{d\tau }$, $\vec{u}\left(r,\tau \right)$ is the cell local displacement field, ${\sigma_{i}}^{CCM}\left(r,t,\tau \right)$ is the cell stress component (normal or shear), ${\sigma_{R\;i}}^{CCM}$ is the cell residual stress, ${{\dot{\sigma }}_{i}}^{CCM}$ is the rate of stress component change ${{\dot{\sigma }}_{i}}^{CCM}=\frac{d}{dt}{\sigma_{i}}^{CCM}$ caused by the stress relaxation,$\;\varepsilon_{i}$ is the cell strain component such that i=1,2,3, while the volumetric strain components 1 and 2 are expressed as $\varepsilon_{1/2}\left(r,\tau \right)=\frac{\partial u_{k}}{\partial k}$, while k≡x,y, and the shear strain $\varepsilon_{3}\left(r,\tau \right)=\frac{1}{2}\left(\frac{\partial u_{k}}{\partial l}+\frac{\partial u_{l}}{\partial k}\right)$**,** while k≡x and l≡y, ${\dot{\varepsilon }}_{i}$ is the corresponding strain rate component equal to ${\dot{\varepsilon }}_{i}=\frac{d}{d\tau }\varepsilon_{i}$, $\eta_{\alpha }$ is the effective modulus, $D^{\alpha }\varepsilon_{i}\;\left(r,\tau \right)=\frac{d^{\alpha }\varepsilon_{i}\left(r,\tau \right)}{d\tau^{\alpha }}$ is the fractional derivative, and α gives the order of fractional derivatives (the damping coefficient). Caputo’s definition of the fractional derivative of a function $\varepsilon_{i}\left(r,\tau \right)$ is used and expressed as $D^{\alpha }\varepsilon_{i}=\frac{1}{Г\left(1-\alpha \right)}\frac{d}{dt}\int_{0}^{\tau }\frac{\varepsilon_{i}\left(r,\tau \prime \right)}{{\left(\tau -\tau \prime \right)}^{\alpha }}d\tau \prime$ (where Г$\left(1-\alpha \right)$ is a gamma function) [55].

Serra-Picamal et al. [21] and Notbohm et al. [31] considered the rearrangement of MDCK cell monolayers as a function of cell packing density $n_{e}\leq n_{conf}$ and revealed that the long-term cell stress (i.e., the cell residual stress) correlates with the corresponding strain, thereby highlighting the viscoelastic solid behaviour. It is known that epithelial cells establish strong E-cadherin-mediated cell-cell adhesion contacts. An additional significant characteristic of epithelial monolayers, particularly relevant to this specific range of cell packing densities, is the ability of cell stress to relax towards the cell residual stress. Khalilgharibi et al. [56] emphasized that stress relaxation time is on a time scale of minutes, while the cell residual stress accumulation occurs on a time scale of hours [53]. According to the findings presented, Pajic-Lijakovic and Milivojevic [53] proposed the scenario that cell stress change occurs through many short-time stress relaxation cycles, while cell strain (caused by collective cell migration) and the corresponding cell residual stress change over a time scale of hours. A suitable constitutive model should satisfy the conditions (1) that the stress relaxes exponentially on a time scale of minutes and (2) that the cell residual stress correlates with the corresponding strain, indicating the long-term elastic behaviour. This model is the Zener model, suitable for viscoelastic solids, as shown in Table 2. In this case, energy dissipation, characteristic of the viscoelastic behaviour of multicellular systems, occurs on a time scale of minutes as a consequence of the remodelling of cell-cell adhesion contacts [20]. The main characteristic of this regime is the pronounced anisotropic behaviour of cell rearrangement. The consequence of this anisotropic viscoelasticity is that cell normal strains in the x- or y-directions contribute to the generation of shear stress, while the shear strain also contributes to the generation of normal and shear stress components. Consequently, shear stress is generated within multicellular domains, as well as along the biointerface between neighbour domains. Serra-Picamal et al. [21] and Tambe et al. [57] revealed that maximum shear and normal, tensional stress components, generated during the free expansion of epithelial monolayers, were about 150 Pa. Notbohm et al. [31] considered the rearrangement of confluent epithelial monolayers and measured compressive stress up to 300 Pa.

An increase in cell packing density, caused by inhomogeneous wetting/de-wetting of migrating epithelial domains, additionally reduces cell movement and perturbs cell alignment as quantified by a decrease in the velocity correlation length. Garcia et al. [33] revealed that a decrease in cell velocity from $\sim 1\;\frac{\mu m}{\min}$ to $\sim 0.1\;\frac{\mu m}{\min}$ results in a decrease in the velocity correlation length from ~150 μm to ~50 μm, and consequently induces the disordering of cell movement. Intensive cell-cell interactions, caused by an increase in cell packing density, are responsive to the transition from convective to conductive mechanisms and can be characterized by an isotropic, linear viscoelasticity. The main characteristic of the conductive regime is that the cell stress cannot relax, while the energy dissipation occurs on a time scale of hours as a consequence of the increased number of cell re-polarisation events. The constitutive model that satisfies these conditions is the Kelvin–Voigt model [53] (Table 2).

An additional rise in cell packing density enhances interactions among cells, which disrupts their alignment and leads to significant orientational interactions between cells. These latter initiate the process of cell re-polarization. Nevertheless, the frequency of cell interactions occurs at a rate that is faster than the time required for re-polarization, resulting in a phenomenon known as jamming (Box 2). In this regime of cell packing densities, collective cell migration occurs via a non-linear sub-diffusion mechanism. The disordering trend of cell migration, quantified by a significant decrease in the velocity correlation length, points to isotropic viscoelasticity, while a non-linear mechanism of cell migration has been described by the Fractional constitutive model (Table 2). The corresponding energy dissipation, caused by perturbation of cell alignment and cell contractile-to-non-contractile transitions in this regime, occurs on a time scale of an hour.

The viscoelasticity of cell monolayers depends primarily on cell contractility and the strength of cell-cell adhesion contacts. Corominas-Murtra and Petridou [58] discussed tissue viscoelasticity as a supracellular network. While epithelial cells form strong E-cadherin-mediated cell-cell adhesion contacts, endothelial cells establish less stable VE-cadherin-mediated cell-cell adhesion contacts [59]. Collective migration of endothelial cells within vascular networks is influenced by shear stress and cell-matrix interactions. In contrast, mesenchymal cells form weaker cell-cell adhesion connections via N-cadherin, resulting in a more dissipative migration pattern compared to that of epithelial cells [14]. Epithelial cancer cells spread through healthy epithelium [18] subsequently invading the stroma and moving towards blood vessels. This process occurs via mesenchymal and amoeboid modes of cell migration, which are influenced mainly by the pore size and viscoelastic properties of the extracellular matrix. The viscoelasticity of cancer-stroma systems depends on the coupling of viscoelasticity of both subsystems, i.e., cancer and matrix. The nature of this coupling is contingent upon the cell-matrix dynamics and the cancer cell’s capacity to degrade the matrix [60]. Human bone marrow mesenchymal stem cells (hBMSCs) are known to exhibit collective migration during osteogenic differentiation and in response to cues from their microenvironment [61]. Self-organizing mineralization dynamics that occur during the differentiation process promote the structural heterogeneity of mesenchymal cells by contributing to localized entropy production [61]. The viscoelasticity caused by the migration of mesenchymal cells has been described by the Maxwell model, which is suitable for viscoelastic liquids [18]. The main characteristics of the Maxwell model are that (i) stress can relax under a constant strain rate and (ii) strain and strain rate cannot relax.

## 4. Physical Parameters That Govern Cell Rearrangement and Entropy Production

Interplay between three physical parameters such as (i) the degree of cell orientation in the direction of cell migration, (ii) cell velocity, and (iii) cell packing density influences the long-term generation of entropy during collective cell migration. Cell packing density and cell velocity changes will be discussed based on formulated force balance and mass balance equations, while the degree of cell orientation will be formulated based on the thermodynamic phase model. All parameters are interconnected. Detailed descriptions of these parameters follow in the next three sections.

### 4.1. The Degree of Cell Orientation in the Direction of Cell Migration

The degree of cell orientation in the direction of cell migration depends on cell-cell positional and orientational interactions (Box 1) and the accumulated cell residual stress (Equations (3) and (4)). A change of this parameter can be expressed in the form of a modified phase model [62,63] as

(6) $$\frac{\partial \left\|\langle \vec{Q}\rangle \right\|\left(r,\tau \right)}{\partial \tau }=\vec{𝜵}\frac{1}{\xi }\left(\left\|\langle \vec{Q}\rangle \right\|\vec{𝜵}\frac{\delta F_{Q}\left(r,\tau \right)}{\delta \left\|\langle \vec{Q}\rangle \right\|}-𝜵\;{\tilde{\sigma }}_{r}\left(r,\tau \right)\right)$$

where $F_{Q}\left(r,\tau \right)$ is the free energy per unit volume caused by orientational cell-cell interactions, ξ is the effective frictional coefficient among cells, and ${\tilde{\sigma }}_{r}\left(r,\tau \right)$ is the cell residual stress accumulation expressed by Equations (3) and (4) and Table 2 for various cell packing density regimes. The free energy can be expressed in the form of a Landau–Ginsburg functional [63] as $F_{Q}=\frac{1}{∆V}\int \left(\frac{k_{1}}{2}{\left\|\langle \vec{Q}\rangle \right\|}^{2}+\frac{k_{2}}{2}{\left|\left\|\langle \vec{Q}\rangle \right\|\right|}^{2}\right)d^{3}r$ (where the parameter $k_{1}$ controls the relaxation of cell alignment, the parameter $k_{2}$ controls the nearest neighbor alignment, and ∆V is an increment of volume of the multicellular system). The accumulation of cell residual stress perturbs the cell alignment [28]. The relaxation time $t_{Q}=\frac{1}{k_{1}}$ corresponds to the cell re-polarisation time and depends inversely on cell packing density, i.e., $t_{Q}\sim {n_{e}}^{-1}$. For cell packing densities near jamming $n_{e}\to n_{j}$, the relaxation time tends to infinity, i.e., $t_{Q}\to \infty$. In accordance with the fact that the cell packing density significantly influences the relaxation time of cell alignment and the accumulation of cell residual stress, it is necessary to formulate the mass balance equation.

### 4.2. The Mass Balance

The change in cell packing density is induced by collective cell migration and the gradient of interfacial tension. Mass balance can be expressed as [40,53]

(7) $$\frac{\partial n_{e}(r,\tau )}{\partial \tau }=\vec{𝜵}{\cdot {\vec{(J}}_{m}}^{e}+{\vec{J}}_{Me})$$

where the cell packing density is $n_{e}\left(r,\tau \right)=\sum_{i}\delta \left(r-r_{i}\left(\tau \right)\right)$ and the flux ${{\vec{J}}_{m}}^{e}$ describes the regime of cell movement (Table 2). For the convective regime, it is the convective flux ${{\vec{J}}_{m}}^{e}\equiv {{\vec{J}}_{conv}}^{e}=n_{e}{\vec{v}}_{e}$, while for the diffusion regime, it is the diffusion flux ${{\vec{J}}_{m}}^{e}\equiv {{\vec{J}}_{diff}}^{e}=-D_{eff}\vec{𝜵}n_{e}$ (where $D_{eff}$ is the effective diffusion coefficient). For the sub-diffusion regime, the corresponding flux ${{\vec{J}}_{m}}^{e}$ represents the sub-diffusion flux ${{\vec{J}}_{m}}^{e}\equiv {{\vec{J}}_{sub-diff}}^{e}$ and the mass balance equation (Equation (7)) should be formulated by including the fractional derivatives, i.e., $D^{\alpha }n_{e}=\vec{𝜵}{\cdot {\vec{(J}}_{dsub-diff}}^{e}+{\vec{J}}_{Me})$, where $D^{\alpha }n_{e}$ is the fractional derivative expressed as $D^{\alpha }\left(n_{e}\right)=\frac{d^{\alpha }n_{e}}{d\tau^{\alpha }}$, and α is the order of the fractional derivative i.e., the damping coefficient of the cell rearrangement near jamming, which satisfies the condition α≤1/2, the flux ${{\vec{J}}_{dsub-diff}}^{e}=-D_{\alpha }\vec{𝜵}n_{e}$, and $D_{\alpha }$ is the damped-conductive diffusion coefficient which has units of $\frac{m^{2}}{s^{\alpha }}$ [53].

The flux ${\vec{J}}_{Me}$ is the Marangoni flux, which depends on the gradient of the epithelial-matrix interfacial tension and has been formulated as ${\vec{J}}_{Me}=k_{Me}n_{e}{\vec{𝜵}}_{s}\gamma_{em}$, where $k_{Me}$ quantifies the mobility of epithelial cells along the biointerface and ${\vec{𝜵}}_{s}\left(\cdot \right)$ is the surface gradient [48]. The Marangoni flux directs the movement of cells from the region of lower interfacial tension toward the region of higher interfacial tension. This phenomenon is observed in numerous soft matter systems, predominantly resulting from variations in temperature and concentration distributions [44].

A change in cell packing density, caused by the generation of mechanical stress, influences energy dissipation and entropy production both directly and indirectly by influencing physical parameters such as the cell velocity and the order parameter. The inter-relationship between cell velocity and other relevant physical parameters will be discussed in the next section in the form of the force balance.

### 4.3. The Force Balance

Oscillations of the driving and resistive forces (Box 3) induce successive establishment and perturbation of dynamical equilibrium, resulting in long-term oscillations in cell velocity. The phenomenon has been observed and discussed in the context of effective inertia [31,40]. The driving forces are the interfacial tension force and the mixing force, whereas the viscoelastic force and traction force constitute the resistive forces. The volumetric force balance can be expressed as [40]

(8) $${\langle m\rangle }_{e}\frac{D\left[n_{e}\left(r,\tau \right){\vec{v}}_{e}\left(r,\tau \right)\right]}{D\tau }={{\vec{F}}_{m}}^{e-m}+n_{e}{{\vec{F}}_{it}}^{e}-{{\vec{F}}_{Tve}}^{e-m}-\rho_{e-m}{{\vec{F}}_{tr}}^{e-m}$$

where $\frac{D\left[n_{e}\left(r,\tau \right){\vec{v}}_{e}\left(r,\tau \right)\right]}{D\tau }$ is the material derivative [64], $\frac{D\left[n_{e}\left(r,\tau \right){\vec{v}}_{e}\left(r,\tau \right)\right]}{D\tau }=n_{e}\left[\frac{\partial {\vec{v}}_{e}}{\partial \tau }+({\vec{v}}_{e}\cdot \vec{𝜵}){\vec{v}}_{e}\right]+{\vec{v}}_{e}\left[\frac{\partial n_{e}}{\partial \tau }+({\vec{v}}_{e}\cdot \vec{𝜵})n_{e}\right]$, which satisfies the condition that $n_{e}\left[\frac{\partial {\vec{v}}_{e}}{\partial \tau }+({\vec{v}}_{e}\cdot \vec{𝜵}){\vec{v}}_{e}\right]\gg {\vec{v}}_{e}\left[\frac{\partial n_{e}}{\partial \tau }+({\vec{v}}_{e}\cdot \vec{𝜵})n_{e}\right]$**,** ${\langle m\rangle }_{e}$ is the average mass of a single cell, ${{\vec{F}}_{mix}}^{e-m}$ is the mixing force, $n_{e}{{\vec{F}}_{it}}^{e-m}$ is the interfacial tension force, ${{\vec{F}}_{Tve}}^{e-m}$ is the viscoelastic force, and $\rho_{e-m}{{\vec{F}}_{tr}}^{e-m}$ is the traction force.

The mixing force ${{\vec{F}}_{mix}}^{e-m}$ arises from the thermodynamic energy changes associated with the mixing of two soft matter systems, specifically the cell monolayer and the substrate hydrogel matrix. This force has been formulated as ${{\vec{F}}_{mix}}^{c-m}=\frac{1}{h_{c}}{\vec{𝜵}}_{s}\left(e_{a}\right)$, where $h_{c}$ is the average size of a single cell. The interfacial tension force $n_{e}{{\vec{F}}_{it}}^{e-m}$ drives cell active/passive wetting and de-wetting. The phenomenon is contingent upon the relationship between the tissue and matrix surface tensions accompanied by the interfacial tension between them. This relationship can be quantitatively represented by the cell spreading factor [40]. This force was formulated as $n_{e}{{\vec{F}}_{it}}^{e-m}=n_{e}S^{e}\vec{u}$, where $S^{e}$ is the spreading factor of epithelial cells and $\vec{u}$ is the epithelial displacement field [40]. The spreading factor for free expansion of cell monolayers satisfies the criteria that $S^{e}>0$ and that change with time causes oscillatory wetting [48].

The traction force is the resistive force formulated by Murray et al. [45] as $\rho_{e-m}{{\vec{F}}_{tr}}^{e-m}=\rho_{e-m}k_{c}{\vec{u}}_{m}$ (where $\rho_{e-m}$ is the number density of cell-matrix adhesion contacts, $k_{c}$ is the spring constant of a single cell-matrix adhesion contact, and ${\vec{u}}_{m}$ is the local displacement of the matrix caused by cell tractions). The establishment of strong cell-matrix adhesion contacts has been shown to diminish cellular motility [64]. The viscoelastic force as formulated by Murray et al. [45] was expressed as ${{\vec{F}}_{ve}}^{e}=𝜵\cdot {(\tilde{\sigma }}_{r}-{\tilde{\sigma }}_{mr})$ (where ${\tilde{\sigma }}_{r}$ is the cell residual stress and ${\tilde{\sigma }}_{mr}$ is the residual stress within a substrate matrix caused by cell tractions). The residual stress within a substrate matrix depends on the matrix viscoelasticity and cell tractions. Cell tractions also influence the matrix surface tension and epithelial-matrix interfacial tension.

The scenario for the oscillatory variation of the velocity ${\vec{v}}_{e}$ is outlined as follows:

- An inhomogeneous accumulation of cell compressive residual stress within the epithelial monolayer caused by collective cell migration leads to an increase in the viscoelastic force [40]. The viscoelastic force and traction force cause a decrease in the velocity of epithelial cells and an increase in the epithelial packing density. An induced decrease in the velocity ${\vec{v}}_{e}$ causes a decrease in the traction force [48].
- An increase in the epithelial packing density leads to intensive cell-cell interactions, resulting in energy dissipation within the epithelial monolayer and, consequently, decreases in (1) the residual stress accumulation within the epithelium; and (2) the epithelial surface tension.
- A reduction in the residual stress present in the epithelial monolayer leads to a decrease in viscoelastic force. As a result, the suppression of both resistive forces, namely the viscoelastic and traction forces, contributes to an increase once more in the velocity of the epithelial cells.
- Consequently, oscillations of cell velocity are thus a result of variations in energy dissipation alongside the generation of entropy.

In addition to examining the long-term dynamics of internal entropy generation, it is essential to address the short-term dynamics of entropy generation within a multicellular domain that is typical of a convective regime.

## 5. Short-Term Dynamics of Entropy Generation Associated with the Convective Regime

The short-term dynamics of entropy generation occur within many successive short-term relaxation cycles and are related to the cumulative effects of the remodelling of cell-cell and cell-matrix adhesion contacts and cell shape changes caused by cell contractions [41]. The average internal entropy of the *m*-th domain established after a single relaxation cycle can be expressed [65] as

(9) $${S_{i}}^{m}\left(t_{eq},\;\tau \right)=-k_{B}N_{d}\sum_{i=1}^{N_{d}}P_{i}lnP_{i}$$

where $N_{d}$ is the number of cells per *m*-th domain and $P_{i}$ is the Boltzmann probability equal to $P_{i}=\frac{e^{-\beta e_{i}}}{Z_{p}}$ (where $Z_{p}$ is the canonical partition function, $e_{i}$ is the mechanical energy of a single cell expressed as $e_{i}=e_{i}\left(q_{1},q_{2},\ldots q_{n}\right)$, while $q_{1},q_{2},\ldots q_{n}$ are the degrees of freedom (DOFs), which describe the phase space of a single cell. Cell activity can be simplified by using a few DOFs such as (i) the cell contractility dimensionless concentration of phosphorylated myosin $q_{1}\equiv \frac{c_{M}}{c_{Me}}$ (where $c_{Me}$ is the equilibrium myosin concentration) [31]; (ii) the dimensionless number density of E-cadherin molecules per single cell $q_{2}\equiv \frac{\rho_{AJ}}{\rho_{AJe}}$ (where $\rho_{AJe}$ is the equilibrium number density of cadherin molecules per single cell); (iii) the dimensionless number density of integrin molecules bonded to ligands of extracellular matrix per single cell $q_{3}\equiv \frac{\rho_{FA}}{\rho_{FAe}}$ (where $\rho_{FAe}$ is the equilibrium number density of integrin molecules per single cell); and (iv) the dimensionless concentration of signalling molecules in the cell surrounding $q_{4}\equiv \sum_{i}\frac{c_{si}}{c_{si0}}$ (where $c_{si0}$ is the equilibrium concentration of the *i*-th signaling molecule), which influences the single-cell state and ordering within a domain [66,67].

A short-time change of the DOFs can be expressed in the form of a system of Langevin-type equations [41] as

(10) $$\frac{dq_{i}\left(t,\tau \right)}{dt}=-\frac{1}{\gamma_{q}\left(\tau \right)}\frac{\partial \langle e\left(q_{1},q_{2},\ldots q_{n}\right)\rangle }{\partial q_{i}}+f_{R}$$

where the stochastic random force $f_{R}$ is formulated as a white noise with correlation function $\langle f_{R}\left(t\right)f_{R}\left(t\prime \right)\rangle =2k_{B}T_{eff}\left(\tau \right)\gamma_{q}\left(\tau \right)\delta \left(t-t\prime \right)$ while $\gamma_{q}\left(\tau \right)$ serves as the equivalent of frictional resistance.

The current equilibrium state after a single short-time relaxation cycle per domain for the time set $\left(t_{eq},\tau \right)$ can be expressed by the canonical partition function $Z_{p}$ as

(11) $$Z_{p}=\int \int \ldots \int \Omega {\;e}^{-\beta E\left(q_{1},q_{2},\ldots q_{n}\right)}dq_{1}dq_{2}\ldots dq_{n}$$

where integration goes over all dimensionless degrees of freedom (DOFs) $q_{i}$ of all cells in the domain and $E\left(q_{1},q_{2},\ldots q_{n}\right)$ is the energetic function equal to $E=N_{d}\langle e\rangle$, $\langle e\rangle$ is the average mechanical energy of single-cell, and $\beta ={{(k}_{B}T_{eff})}^{-1}$.

Consequently, short-term energy production occurs during the stress relaxation cycles within the convective regime.

## 6. Conclusions

- Our theoretical investigation reveals that cells can effectively control entropy generation linked to the dissipation of mechanical energy during collective migration. This collective movement of cells triggers both active and passive wetting and de-wetting on the substrate matrix, leading to an accumulation of mechanical stress within epithelial monolayers. This mechanical stress exerts a feedback influence on various parameters, including cell packing density, alignment, and velocity. Notably, an increase in compressive stress results in an increase in cell packing density, while shear stress can create topological defects in cell alignment, which in turn amplify cell-cell interactions. The principal outcomes of this study were derived from the synthesis of physical models and experimental data in the domains of mechanobiology and biological physics. We can summarize them as follows:
- Cell-cell interactions cause remodelling of cell-cell and cell-matrix adhesion contacts and can induce the re-polarisation of cells accompanied by a weakening of adhesion contacts. These interactions are pivotal in the dynamic alteration of energy dissipation within cells, which is associated with the viscoelastic properties and surface characteristics of multicellular systems, ultimately contributing to entropy generation. The regulation of energy dissipation serves as a cellular mechanism to decrease mechanical stress in epithelial monolayers, thereby inhibiting cell migration.
- Energy dissipation caused by the remodelling of cell-cell and cell-matrix adhesion contacts during relaxation of migrating epithelial collectives under mechanical stress occurs on a time scale of minutes. This phenomenon of entropy production is indicative of a convective regime. The continuous remodelling of adhesion contacts is a fundamental aspect of cellular adaptation to varying microenvironmental conditions and occurs repeatedly throughout the process of collective cell migration.
- Contact inhibition of locomotion, which is pronounced in an overcrowded environment characterized by an increase in cell packing density in a conductive diffusion regime, leads to energy dissipation during the process of cell re-polarization. This re-polarization, accompanied by a weakening of cell-cell and cell-matrix adhesion contacts, occurs over a time frame of several hours. When cells are afforded sufficient time to complete the re-polarization process and re-establish robust adhesion contacts with both neighbouring cells and the extracellular matrix, they initiate movement in the opposite direction.
- When the interval between two collisions is less than the duration required for the re-polarization process, which is typical of the sub-diffusion regime, cells experience jamming. This phenomenon results in a transition from a contractile to a non-contractile state, leading to prolonged energy dissipation and a reduction in mechanical stress. The latter facilitates the formation of robust adhesion contacts between cells and the extracellular matrix, subsequently initiating collective migration once more.
- These oscillations of energy storage and energy dissipation caused by collective cell migration result in oscillations in the production of entropy.

## Figures and Tables

**Table 1:** Surface characterization of epithelial monolayers.

Physical Parameter	Short Description of the Physical Parameters
Spreading factor [43] $S^{e}\left(r,\tau \right)=e_{a}-e_{c}$	$\mathrm{The}\;\mathrm{spreading}\;\mathrm{factor}\;\mathrm{of}\;\mathrm{epithelial}\;\mathrm{monolayers}\;\mathrm{is}\;\mathrm{related}\;\mathrm{to}\;a\;\mathrm{difference}\;\mathrm{between}\;\mathrm{cohesion}\;\mathrm{and}\;\mathrm{adhesion}\;\mathrm{energy}.\;\mathrm{When}\;\mathrm{the}\;\mathrm{spreading}\;\mathrm{factor}\;\mathrm{is}\;S^{e}\left(r,\tau \right)>0$ $,\;\mathrm{epithelial}\;\mathrm{cells}\;\mathrm{undergo}\;\mathrm{wetting},\;\mathrm{while}\;\mathrm{for}\;S^{e}\left(r,\tau \right)<0$, epithelial cells undergo de-wetting [19,43].
Epithelial-matrix adhesion energy [45] $e_{a}\left(r,\tau \right)=\rho_{e-m}\frac{1}{2}k{\left|{\vec{u}}_{m}\right|}^{2}$	$\mathrm{Epithelial}-\mathrm{matrix}\;\mathrm{adhesion}\;\mathrm{energy}\;\mathrm{depends}\;\mathrm{on}\;\mathrm{the}\;\mathrm{density}\;\mathrm{of}\;\mathrm{adhesion}\;\mathrm{contacts}\;\rho_{e-m}$ , the strength of the integrin bond quantified by the spring constant k $,\;\mathrm{and}\;\mathrm{the}\;\mathrm{matrix}\;\mathrm{displacement}\;\mathrm{field}\;\mathrm{caused}\;\mathrm{by}\;\mathrm{cell}\;\mathrm{tractions}\;{\vec{u}}_{m}$.
Epithelial cohesion energy is the energy necessary for the separation of two previously connected epithelial surfaces and can be expressed as $e_{c}\left(r,\tau \right)=2\gamma_{e\;}$ $\mathrm{where}\;\gamma_{e}$ is the epithelial surface tension	$\mathrm{The}\;\mathrm{epithelial}\;\mathrm{surface}\;\mathrm{tension}\;\gamma_{e}$ serves as an indicator of the cohesiveness of epithelial tissue when in contact with a liquid medium. $\mathrm{This}\;\mathrm{parameter}\;\mathrm{quantifies}\;\mathrm{the}\;\mathrm{change}\;\mathrm{in}\;\mathrm{surface}\;\mathrm{energy}\;\mathrm{of}\;a\;\mathrm{multicellular}\;\mathrm{surface}\;E_{S}$ $\mathrm{by}\;\mathrm{changing}\;\mathrm{the}\;\mathrm{surface}\;\mathrm{area},\;\mathrm{and}\;\mathrm{can}\;\mathrm{be}\;\mathrm{expressed}\;\mathrm{as}\;\gamma_{e}=\frac{\partial E_{S}}{\partial A}$ (where A is the surface area). The surface energy accounts for three contributions: (i) the elastic contribution caused by a change in the surface area of cells, (ii) the contribution of cell-cell adhesion contacts, and (iii) the contractile energy of cells [46,47].
Matrix surface tension $\gamma_{m}$ $\left(r,\tau \right)$	Matrix surface tension depends on inter- and intra-chain interactions and is influenced by cell tractions [48].
Epithelial-matrix interfacial tension [40] $\gamma_{em\;}\left(r,\tau \right)=\gamma_{e\;}+\gamma_{m}-e_{a}$	Epithelial-matrix interfacial tension does work in reducing the biointerface area, thus generating the isotropic part of the cell compressive stress [19].
Gradient of epithelial-matrix interfacial tension [40] ${\vec{𝜵}\gamma }_{em}\left(r,\tau \right)$	The gradient of the epithelial-matrix interfacial tension directs cell movement from regions of lower interfacial tension to regions of higher interfacial tension by leading to passive wetting/de-wetting. The phenomenon has been known as the Marangoni effect [44]. The experimental validation of cell migration across multicellular surfaces, driven by gradients in surface tension, was established by Gsell et al. [49].

**Table 2:** Some constitutive models proposed for various modes of epithelial cell migration.

Mechanism of Epithelial Cell Migration	Constitutive Models for the Viscoelasticity of Epithelial Monolayers	Energy Dissipation
(1) Convective regime (anisotropic linear viscoelasticity) ${\left\|\langle \vec{Q}\rangle \right\|}_{1}\to 1$ $n_{e}\leq n_{conf}$ $<\left\|{\vec{v}}_{e}\right\|<\sim 1\;\frac{\mu m}{\min}$	The Zener model for viscoelastic solids: ${\sigma_{i}}^{CCM}\left(r,t,\tau \right)+\tau_{Rc}\;{{\dot{\sigma }}_{i}}^{CCM}=\sum_{j=1}^{3}c_{ij}\varepsilon_{j}+\eta_{ij}{\dot{\varepsilon }}_{j}$ Stress relaxation under constant strain conditions per single short-time relaxation cycle: ${\sigma_{i}}^{CCM}\left(r,t,\tau \right)={\sigma_{0i}}^{CCM}e^{-\frac{t}{\tau_{Rc}}}+{\sigma_{Ri}}^{CCM}\left(r,\tau \right)\left(1-e^{-\frac{t}{\tau_{Rck}}}\right)$ Cell residual stress is elastic. ${\sigma_{Ri}}^{CCM}=\sum_{j=1}^{3}c_{ij}\varepsilon_{0j}$	${W_{d}}^{CCM}\left(r,t,\tau \right)=\sum_{i=1}^{3}∆{\sigma_{i}}^{CCM}\left(r,t,\tau \right)\varepsilon_{j}\left(r,\tau \right)$ where the stress difference is $\mathrm{the}\;\mathrm{mechanical}\;\mathrm{stress}\;\mathrm{difference},\;\mathrm{equal}\;\mathrm{to}\;∆{\sigma_{i}}^{CCM}\left(r,t,\tau \right)={\sigma_{i}}^{CCM}\left(r,t,\tau \right)-{\sigma_{iR}}^{CCM}\left(r,\tau \right)$ Average energy dissipation is ${\langle W_{d}\rangle }^{CCM}\left(r,\tau \right)=\frac{1}{∆t}\int_{0}^{∆t}\;W_{d}\left(r,t,\tau \right)\;dt$ where ∆t is the time necessary for stress relaxation.
(2) Diffusion regime (isotropic linear viscoelasticity) ${\left\|\langle \vec{Q}\rangle \right\|}_{2}\ll {\left\|\langle \vec{Q}\rangle \right\|}_{1}$ $n_{j}>n_{e}>n_{conf}$ $\left\|{\vec{v}}_{e}\right\|\sim 10^{-3}-10^{-2}\frac{\mu m}{\min}$	The Kelvin-Voigt model for viscoelastic solids: ${\sigma_{i}}^{CCM}\left(r,\tau \right)=E_{i}\varepsilon_{i}+{\eta_{i}\dot{\varepsilon }}_{i}$ $\mathrm{where}\;E_{1}\approx E_{2}$ $\mathrm{and}\;\eta_{1}\approx \eta_{2}$, while the viscous part of the stress is ${\sigma_{vis\;i}}^{CCM}\left(r,\tau \right)=\eta_{i}{\dot{\varepsilon }}_{i}\left(r,\tau \right)$ and its elastic part ${\sigma_{el\;i}}^{CCM}\left(r,\tau \right)=E_{i}{\tilde{\varepsilon }}_{i}$ The stress cannot relax. ${\sigma_{i}}^{CCM}={\sigma_{R\;i}}^{CCM}$	${W_{d}}^{CCM}\left(r,\tau \right)=\sum_{i=1}^{3}{\sigma_{vis\;i}}^{CCM}\varepsilon_{i}$
(3) Sub-diffusion regime (isotropic nonlinear viscoelasticity) ${\left\|\langle \vec{Q}\rangle \right\|}_{3}\to 0$ $n_{e}\to n_{j}$ $\left\|{\vec{v}}_{e}\right\|\to 0$	The fraction model for viscoelastic solids: ${\sigma_{i}}^{CCM}\left(r,\tau \right)=\eta_{\alpha }D^{\alpha }\left(\varepsilon_{i}\right)$ For 0<α<1/2 Elastic and viscous parts of stress cannot be dissociated [54]. The stress cannot relax. ${\sigma_{i}}^{CCM}={\sigma_{R\;i}}^{CCM}$	${W_{d}}^{CCM}\left(r,\tau \right)=W_{d}\left(\alpha ,\eta_{\alpha }\right)$

## Data Availability

No new data were created in this study. Data sharing is not applicable to this article.
